# Recognition and Treatment of Cognitive Dysfunction in Major Depressive Disorder

**DOI:** 10.3389/fpsyt.2018.00655

**Published:** 2018-12-04

**Authors:** Hannah Zuckerman, Zihang Pan, Caroline Park, Elisa Brietzke, Natalie Musial, Aisha S. Shariq, Michelle Iacobucci, Samantha J. Yim, Leanna M. W. Lui, Carola Rong, Roger S. McIntyre

**Affiliations:** ^1^Mood Disorders Psychopharmacology Unit, University Health Network, Toronto, ON, Canada; ^2^Institute of Medical Science, University of Toronto, Toronto, ON, Canada; ^3^Department of Psychiatry, Federal University of São Paulo, São Paulo, Brazil; ^4^Department of Pharmacology, University of Toronto, Toronto, ON, Canada; ^5^Department of Psychiatry, University of Toronto, Toronto, ON, Canada; ^6^Brain and Cognition Discovery Foundation, Toronto, ON, Canada

**Keywords:** cognition, cognitive dysfunction, major depressive disorder, functionality, treatment

## Abstract

Major Depressive Disorder (MDD) is a prevalent, chronic, disabling, and multidimensional mental disorder. Cognitive dysfunction represents a core diagnostic and symptomatic criterion of MDD, and is a principal determinant of functional non-recovery. Cognitive impairment has been observed to persist despite remission of mood symptoms, suggesting dissociability of mood and cognitive symptoms in MDD. Recurrent impairments in several domains including, but not limited to, executive function, learning and memory, processing speed, and attention and concentration, are associated with poor psychosocial and occupational outcomes. Attempts to restore premorbid functioning in individuals with MDD requires regular screenings and assessment of objective and subjective measures of cognition by clinicians. Easily accessible and cost-effective tools such as the THINC-integrated tool (THINC-it) are suitable for use in a busy clinical environment and appear to be promising for routine usage in clinical settings. However, antidepressant treatments targeting specific cognitive domains in MDD have been insufficiently studied. While select antidepressants, e.g., vortioxetine, have been demonstrated to have direct and independent pro-cognitive effects in adults with MDD, research on additional agents remains nascent. A comprehensive clinical approach to cognitive impairments in MDD is required. The current narrative review aims to delineate the importance and relevance of cognitive dysfunction as a symptomatic target for prevention and treatment in the phenomenology of MDD.

## Introduction

Major Depressive Disorder (MDD) is a prevalent, often chronic, and highly disabling multidimensional psychiatric illness affecting ~350 million individuals worldwide (1). Epidemiological research suggests that depression constitutes the leading cause of disability worldwide (1). MDD is characterized by short- and/or long-term impairment affecting areas including, but not limited to, mood, affect, motivation, and cognition, and is frequently correlated with significant reductions in quality of life and psychosocial functioning (2). Despite therapeutic advances in MDD, an estimated 70% of patients do not achieve remission following first-line antidepressant medication (3). Moreover, a significant percentage of patients that successfully achieve conventional symptomatic remission (i.e., reduction in total depressive symptom severity) do not return to premorbid functioning (4). The lack of full functional recovery results in decreased workplace functionality and productivity, further contributing to the significant economic burden imposed by MDD, with an accumulated annual loss of $43 billion in North America (5, 6).

Several regions of the brain, including the hippocampus, are negatively implicated in the pathophysiology MDD. Hippocampal size has been demonstrated to be inversely correlated with illness duration, whereby smaller hippocampal sizes have been associated with more severe histories of depression (7). Size has also been shown to be influenced by the number of past hospitalizations and recurrence of the disorder. Frequent, chronic and lengthy states of depression impart impairing effects on brain function, debasing human capital. Without adequate treatment, brain recovery may be compromised, resulting in negative downstream effects on the global functional outcomes in MDD. Amongst the disparate domains affected in depression, cognition is the most relevant dimension related to the loss of human capital. While MDD treatment efforts have focused on clinically observed symptomatic targets including depressed mood and anhedonia, emerging evidence has dissociated symptomatic improvement in these domains from functional improvement and timely return-to-work (8). The foregoing transition highlights the necessity for novel targets more closely associated with the restoration of premorbid psychosocial functioning. Toward this aim, cognitive dysfunction has emerged as a key mediator subserving adverse functional impairment in MDD (9–12).

Cognition is a nons-pecific term that refers to mental processes associated with thinking, learning, and memory (13). Cognitive dysfunction can be defined as a transnosological domain serving as an essential mediator of disparate mental disorders (14). Clinical presentation of MDD, as defined by the Diagnostic and Statistical Manual of Mental Disorders-Fifth Edition (DSM-5), includes cognitive impairment as a criterion item of a Major Depressive Episode (MDE) (15). Self-reported measures of diminished concentration and attention are frequently observed in individuals presenting with MDE as part of MDD. Moreover, when treating MDD, cognitive impairments are often found to persist during periods of symptomatic remission (14), supporting the disconnect between emotional and functional improvement. While cognitive symptoms may indirectly improve as a consequence of standard antidepressant care, cognitive dysfunction is believed to be a core disturbance in subsets of adults with MDD, independent of mood symptoms (16).

MDD fundamentally alters one's perception and interaction with their surrounding environment, affecting not only the social environment but also information and intellectual processing. Insufficiency surrounding remission outcomes amongst individuals with MDD delineates the importance of novel identification and optimization in recognition and treatment avenues. Successful outcomes rely on a heightened focus encompassing the needs of the patient, the provider, and societal perspectives. Recent developments have thus begun to uncover the relevance of cognition as a clinical priority. Hitherto, disturbances in cognitive function have been undermined in their significance in MDD relative to other psychiatric disorder populations, however, accumulating evidence indicates that a disturbance in cognitive function represents a principal determinant of health outcomes in subsets of MDD patients. Cognitive dysfunction in MDD is common, pervasive across multiple subdomains of cognitive function, and provides a principal determinant of health outcomes with respect to the patient, as well as the societal perspectives. Herein, the current narrative review will provide an up-to-date summary of the literature pertaining to the domain of cognitive function in MDD. The current review aims to provide a framework for the conceptualization of cognition in MDD, with particular focus on the relevance, measurements, and treatment strategies explicating depression as a progressive cognitive disorder.

## Methods

The authors conducted a narrative review of studies investigating cognition as a relevant aspect of Major Depressive Disorder. Studies were identified using PubMed/Medline and Google Scholar from inception to June 2018. *MDD* (and/or variants) was cross-referenced with the following search terms: *cognition, cognitive dysfunction, cognitive deficit, cognitive function, functional outcomes, antidepressants*, and *treatment*. Articles informed by observational studies, clinical trials, and review articles relevant to cognition and cognitive impairment in MDD were included. Additionally, the search was augmented through manual review of related terms and citations from article reference lists.

### Domains of Cognition

Cognition and emotion are interconnected processes originating from large interacting networks of neurons within the brain. In recent decades, interdisciplinary fields including neurophysiology, and cognitive psychology have shed light on the neural underpinnings of various cognitive functions and processes; however, the current understanding of these phenomena remains rudimentary. Cognition is multidimensional and lacks a singular consensually agreed upon taxonomy. Several typologies have been proposed for the definition and operationalization of cognitive constructs. Amongst these is the conventional typology distinguishing cognitive aspects into four main domains—namely executive function, attention/concentration, learning/memory, and processing speed (17). The aforementioned domains are interconnected yet distinct phenomena. An additional proposed typology was introduced by the RDoC which, although not limited to cognitive domains, it emphasizes disturbances across multiple cognitive subdomains (18, 19). Moreover, the literature has further proposed a clinically relevant taxonomy with two distinct domains—namely, “cold” cognition and “hot” cognition (20) (Table 1). By definition, “cold” cognition refers to non-emotional information processing; therefore, these cognitive processes occur in the absence of emotional engagement and/or motivation. “Cold” cognitive processes are used in the evaluation of neuropsychological function in depression (20) and generally include the following subdomains of cognition: executive function, learning and memory, attention and concentration, and processing speed. Examples of commonly administered objective neuropsychological tests include the Rey Auditory Verbal Learning Test (RAVLT) (e.g., acquisition and recall), Trail-Making Test A/B (TMT A/B TMT A: processing speed TMT B: processing speed, executive function i.e., set shifting), and Digit Symbol Substitution Test (DSST processing speed, executive function, learning and memory, attention, and concentration) (12). In contrast, “hot” cognition refers to emotionally-laden cognitive processes; these functions are influenced by the individual's emotional state and may include negative attentional bias, emotionally-linked recall, rumination, and anhedonia (20). However, it is important to note that the weighted significance and distinction between “hot” and “cold” cognition in depressed individuals is non-discrete and there are many overlapping features between the two constructs (20, 21).

**Table 1:** Hot and cold cognitive processes.

**Hot cognition**	**Cold cognition**
Rumination	Executive function
Emotional processing	Processing speed
Anhedonia (reward processing)	Learning and memory
Attentional bias	Attention and concentration

Neuropsychological testing reveals important inferences into disruptive pathophysiology of neural brain networks with direct consequences on “cold” cognitive functioning. Neural networks including the prefrontal cortex and cingulate gyrus, subcortical regions in the striatum and thalamus, and temporal lobe structures including the amygdala and hippocampus are found to be functionally altered in depressive states (22). More specifically, deficits in executive functioning have been associated with pathophysiology in the lateral aspects of the prefrontal cortex. Additionally, memory impairment has been evidenced to be associated with reductions in hippocampal volume which may be a progressive consequence of MDD (22). The circuitry of these structures has formed the targeted basis of various established treatments for depression, however, currently available treatments are not effective in all cases and requires further understanding into the cognitive deficits and neural markers characterizing MDD.

### MDD as a Cognitive Disorder

Cognitive deficits in MDD are consistent, replicable, non-specific, and clinically significant. As cognition comprises an important phenomenological domain of MDD, abnormalities in cognition may be used as a prognostic indicator for identifying at-risk individuals and/or assessing disease onset and progression. Manifestation of cognitive deficits are heterogeneous across individuals with MDD and vary depending on disparate individual- and illness-specific factors. For example, the magnitude of cognitive deficits has been demonstrated to be proportionate to the frequency of depressive episodes and duration of illness (7, 12). In keeping with this, individuals with greater depressive symptom severity are more likely to present with cognitive impairments as compared to those with milder illness severity (12). Moreover, a systematic review evaluating clinical progression in affective disorders, including MDD, suggested that cognitive function is associated with the duration and number of prior episodes (23). Unipolar depression has also been found to be associated with an increased risk of developing dementia, commonly understood as the end stage of progression of cognitive disturbances (23).

**Figure 1:**
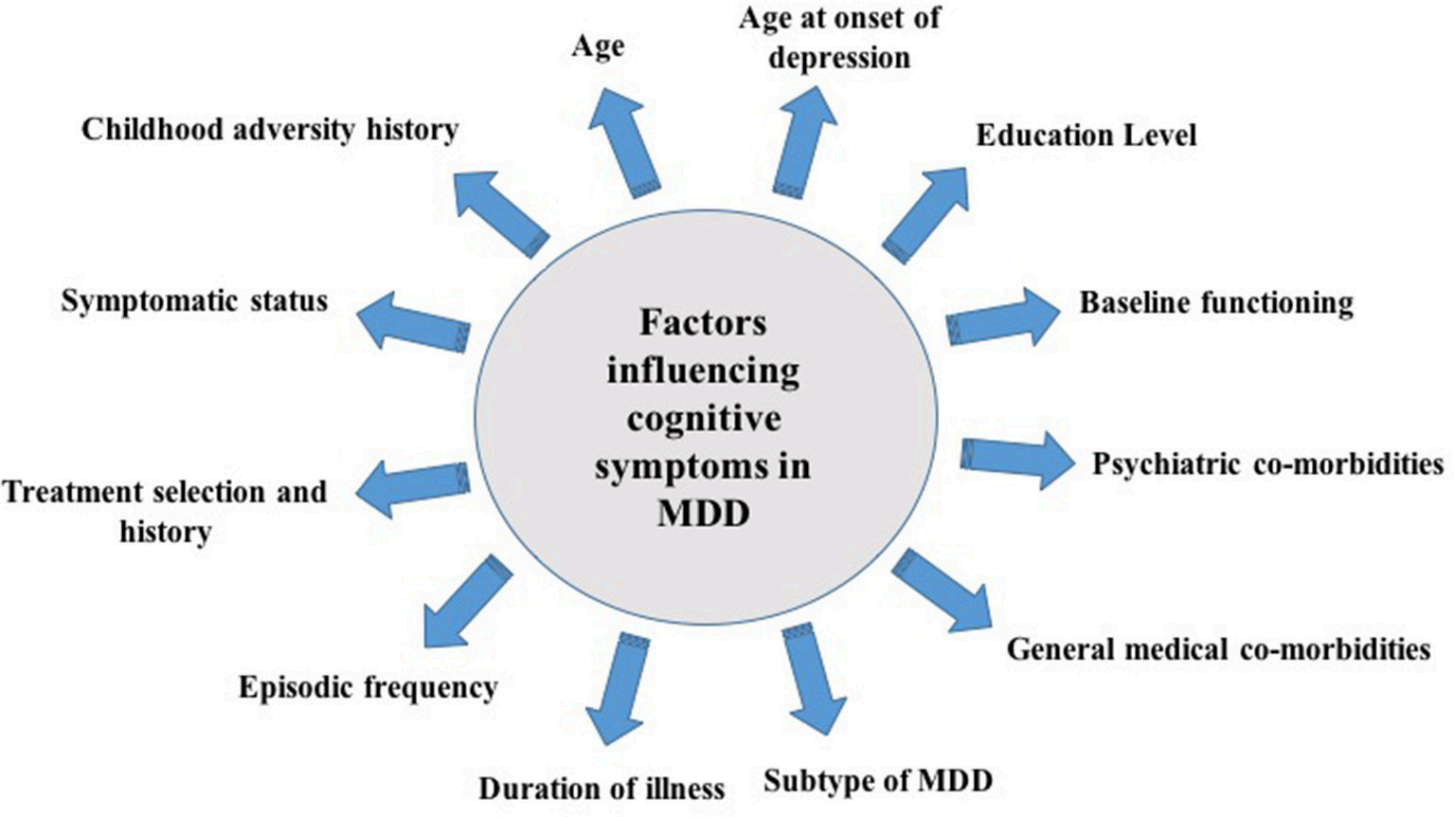
Factors that influence cognitive symptoms in Major Depressive Disorder (MDD).

It is important to note that available studies often include highly heterogeneous populations. This is an important consideration as there are various co-determinants of cognitive function in MDD that may exact mediational and/or moderational effects alongside illness severity and duration. For example, the presence of co-morbid medical and/or psychiatric conditions may exert direct effects on cognitive function and performance (24, 25). Metabolic co-morbidities, such as obesity, have also been associated with cognitive impairments and are commonly observed in depressed individuals (26). In particular, studies have suggested that obesity is correlated with significant deficits in executive functions such as working memory, planning, and executive control (24). In addition, factors such as age (27), age at onset of depression (28), level of education (29), MDD subtype (30), inflammatory status (31), treatment regimen (32), and childhood adversity (33) have also been demonstrated to influence cognitive performance in patients presenting with MDD (Figure 1). Significant impairments in cognitive domains have been reported to precede, occur during, and follow an illness episode; therefore, the temporality and/or causality of the association between cognitive impairment and MDEs remains elusive. Although scarce, studies have evaluated cognitive dysfunction as a risk factor for the development of MDD. In a population study evaluating non-depressed individuals between 20 and 64 years of age found that low episodic memory performance was a reliable predictor of depression 3 years post-diagnosis (34). Moreover, a separate study evaluating longitudinal profiles of depressive symptoms in a birth cohort found that depression is associated with neurodevelopmental impairments which may be mediated by cognition (35). Moreover, cognitive functioning may also be used as a predictor of treatment response. In a recently conducted systematic review of studies evaluating early cognitive change as a predictor of treatment response in individuals with MDD (*n* = 7), early changes in cognitive functioning were demonstrated to have a predictive effect on treatment response. More specifically, the results denoted a trend toward early changes in hot cognitive processes (i.e., changed in facial emotion recognition) as a predictor of response in MDD pharmacotherapy (36).

Clinically, cognition has been classified into four subdomains: (1) learning and memory, (2) attention and concentration, (3) executive function, and (4) processing speed. Patients presenting with MDD commonly experience impairments in each of the principal subdomains of cognition, with ~50% of patients exhibiting deficits of greater than one standard deviation (*SD*) below the mean and 48% of patients two *SD*s below the mean in at least one subdomain (37). Notwithstanding, these reported measures are limited in that they do not necessarily take into consideration subjective cognitive deficits; for example, they may underreport deficits in those individuals whose cognitive performance remains above the mean, but who report deficits in comparison to their baseline level of cognitive function. While MDD does not decrease overall measures of intelligence, cognitive performance across the aforementioned domains have been shown to be severely affected, with effect sizes ranging from 0.2 to 0.8 (14, 38).

### Cognitive Impairment in MDD and its Role in Psychosocial Workplace Functioning

Cognitive impairment has been reported to affect function independent of mood symptoms, and has been correlated with functional impairments. The evidence suggests that select symptom domains, such as cognition, may be of greater relevance to overall health outcomes (39). Neurocognitive deficits in this population are directly related to impaired workplace performance, and have significantly contributed to the overall costs associated with depressive illness (12). Individuals with moderate to severe depression have been demonstrated to experience increased rates of unemployment, disability, and absenteeism from work (40). Reductions in workplace productivity and performance has been shown to be mediated by cognitive impairments. For example, one study that evaluated the relationship between depression and role functioning using a population survey found that impairments in attention and concentration mediated the association between depression and impaired role functioning (9). In addition, a *post-hoc* analysis of data from 260 participants enrolled in the International Mood Disorders Collaborative Project (IMDCP) found that in a subpopulation of working adults (18–65 years of age) diagnosed with MDD, cognitive function was a greater determinant of overall workplace performance than total depression symptom severity (41). These observations suggest that cognitive dysfunction is a principal mediator of functional impairment and highlights their relevance in the evaluation and management of outcomes in MDD.

Replicated evidence indicates that disturbances in cognitive function are common both during, and residually following, an acute MDE (12, 42). Moreover, despite depressive symptom remission, individuals have reported continued deficits in cognitive function, which have negative effects on global function, workplace productivity/performance and quality of life (41). For example, a study found that patients who were currently in a state of remission and who met ICD-10 criteria for former MDD experienced persistent cognitive deficits compared to age-, gender-, and education-matched control subjects (42). Although continued research is required to determine which cognitive deficits persist following mood remission, there appears to be documented deficits in the domains of attention and executive performance when compared to healthy controls (42–44).

### Measurement and Screening of Cognitive Function

Hitherto, a comprehensive “gold standard” measure of cognitive function in MDD with broad conceptual coverage, sensitivity to change, and immune from practice effects does not yet exist. Limitations of conventional clinical assessment measures [e.g., Hamilton Depression Rating Scale (HAMD), MADRS] are suboptimal insofar as they contain insufficient items assessing cognitive function. Moreover, they are subjective in nature and have been shown to have minimal correlation with objective measures of cognition (39). Subjective cognition is often influenced by emotional state, and consequently may be affected by the severity of depressive symptoms to a greater extent than objective measures of cognition. Therefore, although subjective measures provide an accurate report of *perceived* cognitive function, it does not necessarily formulate an accurate measure of objective cognitive ability. In clinical practice, a vast array of neurocognitive tests have been employed for the measurement and evaluation of cognitive impairments in MDD [e.g., DSST, TMT, Perceived Deficits Questionnaire (PDQ)]. These conventional instruments are routinely used and frequently administered for the assessment of cognitive function; however, they were not specifically developed or tailored to evaluate MDD-specific cognitive deficits.

Limitations related to the accessibility, cost-effectiveness, and ecological validity of standard tools for measuring cognition in MDD need to be addressed for the improvement of health outcomes in MDD. The THINC-integrated tool (THINC-it) is a recently validated, computerized cognitive assessment battery that screens both objective and subjective cognitive deficits in MDD (45). The THINC-it tool was validated as a sensitive tool in detecting and quantifying the magnitude of cognitive deficits in adults between the ages of 18 and 65 with MDD (45). It effectively evaluates objective measures of cognition through the inclusion of adaptations of four validated tests (i.e., N-Back/Symbol Check, DSST/Codebreaker, TMT-B/Trails, and CRT/Spotter). These tasks accurately assess cognitive subdomains including working memory, visuospatial coordination, set shifting, and psychomotor speed. Additionally, subjective measures of cognition are evaluated through the Perceived Deficits Questionnaire (PDQ-5). Compared to traditional pen-and-paper based cognitive measures such as the DSST, TMT-B and PDQ-5-D, the tool has greater temporal reliability and concurrent validity (45). It is important to note, however, that there are not clear cut-off criteria or precise values for the individual tests that would enhance its use in both diagnosis and treatment response clinically. To our knowledge, the THINC-it is the first freely available computerized tool for screening cognitive dysfunction in MDD. Other developed methods for screening cognitive dysfunction in MDD include the Screen for Cognitive Impairment in Psychiatry (SCIP-D) and the Cognitive Complaints in Bipolar Disorder Assessment (COBRA). The SCIP-D and COBRA have been validated as methods to assess objective and subjective cognitive impairment in MDD, respectively (46). While the COBRA has poorer sensitivity and specificity for the detection of objective dysfunction as compared to the SCIP-D, the SCIP-D has greater sensitivity and specificity for objective dysfunction; therefore, the COBRA can be used in combination with the SCIP-D to increase sensitivity and specificity for the detection of objective dysfunction in MDD (46).

### Treatment Interventions Targeting Cognitive Dysfunction in MDD

The evaluation of subsyndromal depressive symptoms (SSD) in patients with MDD is relevant for both treatment selection and outcome. The presence of SSD has been shown to be significantly associated with disability and functional impairments in older adults (47). The pertinence of cognitive dysfunction in MDD underscores the critical importance of developing treatment modalities that are capable of directly and/or indirectly improving cognitive function. Classical pharmacological antidepressant therapy aims to achieve symptomatic remission by targeting mood symptoms; however, residual impairments in cognition that are not sufficiently targeted may impose negative effects on workplace performance and productivity or delay a timely return to work. Targeted treatment of cognitive impairments in MDD may capitalize on modifiable determinants, focusing on prevention and pre-emption.

#### Cognitive Remediation and Cognitive Therapy

Psychological methods have been proposed for the treatment and management of neurocognitive impairments in disparate neuropsychiatric conditions including MDD (48). Brain imaging evidence in MDD reveals a pattern of increased activity in the limbic system coincident with decreased activity in executive areas of the brain (49). Cognitive remediation (CR) is a psychosocial approach aimed at relieving cognitive impairments in individuals with diverse brain disorders including, but not limited to, attention deficit hyperactivity disorder (ADHD), autism spectrum disorder (ASD) and schizophrenia. Cognitive remediation involves the use of behavioral strategies to exert a beneficial effect across a broad range of functionally-relevant domains (e.g., psychosocial skills). It has been used for the treatment of maladaptive cognitive thought patterns that are prevalent in MDD. Cognitive training has effects on brain structure and function, and has demonstrated effects on neurobiological systems adversely affected in mood disorders such as schizophrenia) (50). Although strong empirical evidence exists for the use of CR in patients with schizophrenia, few studies have been conducted evaluating the efficacy of CR in homogeneous samples of unipolar depressed individuals. When used as an adjunctive therapy in patients with MDD (*n* = 12), CR was found to significantly improve cognitive function (i.e., attention, verbal learning and memory, psychomotor speed, and executive function) compared to patients who were not receiving adjunctive CR (50). The neuropsychological educational approach to remediation (NEAR) has been used as a method of CR that focuses on aspects of motivation and learning. NEAR involves the delivery of individually-tailored and commercially available computer games in a group setting; the games are tailored to an individual's strengths and weaknesses to promote learning via positive reinforcement (51). Significant improvements in cognitive function, particularly verbal memory, have been found in patients with MDD receiving NEAR in addition to routine therapy, as compared to patients receiving only routine therapy R. S. C. (51–53). Although theoretically promising, more evidence is required to suggest that CR is effective for the long-term treatment of cognitive dysfunction in MDD (14). Additional considerations for the improvement and validity of CR for the treatment of cognitive dysfunction in MDD include consistency with regard to task selection (i.e., degree of difficulty across studies), treatment frequency, and generalizable measurements.

Cognitive behavior therapy (CBT) is a well-studied and propitious therapeutic avenue for the treatment and management of cognitive dysfunction in MDD. The use of CBT in combination with pharmacotherapy in adult depression has been shown to target primarily hot cognitive processes and be more effective than pharmacotherapy alone (54). Notwithstanding, there are a number of patients that do not successfully respond to CBT (55). Consequently, studies have aimed to investigate various subtypes and pathoetiology of depression with the goal of informing possible predictors of treatment response. Several studies have suggested that abnormalities in medial prefrontal cortex (MPFC) (56) and the anterior cingulate cortex (ACC) (57) may contribute to the cognitive impairments observed in MDD. For example, a study found that following CBT treatment, the functional connectivity between MPFC-ACC was significantly reduced. Moreover, symptomatic improvement was positively correlated with a change in MPFC-ACC functional connectivity, (58) suggesting that CBT could potentially be effective as a precognitive intervention.

#### Neurostimulation

Neurostimulation methods have been shown to be highly effective in the treatment of depression. For example, electroconvulsive therapy (ECT) is a neurostimulation method that has been demonstrated to be efficacious in the acute treatment of depression (59). Negative neurocognitive bias represents a centric feature of major depression that is associated with significant risk of relapse (60), with evidence highlighting negative face processing persisting into periods of remission (61). Previous research provides evidence for various antidepressants as rapid modulators of cognitive and neural emotional face processing, prior to symptom improvement (62, 63). Contrary to this, compounds lacking antidepressant efficacy have no effect on emotional bias (63). The capacity of ECT as a modulator of neurocognitive response to emotional information has recently been studied. In the first conducted double-blind, sham-controlled, parallel-group study, the effect of a single ECT session on the neurocognitive response to emotional information in MDD was evaluated. The results revealed changes in parahippocampal and superior frontal responses to fearful vs. happy faces, as well as in fear-specific functional connectivity between amygdala and occipito-temporal regions in response to single-session ECT treatment (64). Although no statistically significant shift in the neural response to faces was observed following ECT, the trend is suggestive of early shifts in emotional processing that contribute to the antidepressant activity of ECT (64).

Despite its promising use in the reversal of heightened neurocognitive response in MDD, the use of ECT is prejudiced due to reports of cognitive impairments following individual treatment. Some patients report acute disorientation and cognitive deficits, with uncertainty surrounding the duration of the short- and long-term cognitive deficits. In a meta-analysis of 84 studies and 2,981 patients that aimed to evaluate the cognitive impairments following ECT, significant cognitive impairments (i.e., in verbal and visual episodic memory and executive function) were identified in depressed patients within 3 days of treatment. However, these deficits in cognitive function following ECT did not persist beyond 15 days post-treatment, with small to medium effect sizes of improvement for most variables (i.e., processing speed, verbal working memory, and executive function)(59).

More recently, repetitive transcranial magnetic stimulation (rTMS) has emerged as a neurostimulation method for improving neurocognitive function in patients with MDD, and is generally viewed more favorably due to its less invasive nature and diminished propensity for cognitive impairment (65). A systematic review analyzing the role of rTMS in improving neurocognition in patients with treatment-resistant depression (TRD) found that the majority of studies support an association between rTMS and improved neurocognitive effects (66). Much of the literature focuses on the dorsolateral prefrontal cortex (DLPFC) as a target for rTMS as it is a critical brain region for neurocognitive performance (67). Stimulation of the DLPFC through rTMS has been shown to produce variable improvements in psychomotor speed, attention, verbal fluency, executive function, and other working memory domains in patients with TRD (66). The foregoing evidence suggests potential procognitive effects following neurostimulation via rTMS. However, it remains to be determined whether rTMS and/or other neurostimulation techniques can reliably and effectively ameliorate cognitive dysfunction in MDD.

#### Pharmacotherapy

The counterintuitive gap between remission from depressive symptoms and functional recovery warrants the evaluation of therapeutic avenues targeted at improving cognitive symptoms in individuals with MDD. Notwithstanding the current need for novel targets to facilitate symptomatic remission coincident with functional productivity and timely return-to-work, pharmacotherapies have been scarcely evaluated for their direct and independent effects on cognition in MDD. Individuals with MDD routinely receive interventions [e.g., selective serotonin reuptake inhibitors (SSRIs), serotonin and norepinephrine reuptake inhibitors (SNRIs), monoamine oxidase inhibitors (MAOIs), antipsychotics] with potentially adverse effects on cognitive measures (20). Persistent functional impairments mediated by cognitive dysfunction in individuals with MDD warrants the identification of pharmacotherapies with pro-cognitive effects.

Determination of the magnitude of effect of an antidepressant on cognitive function has posed significant limitations as study designs and assessment methodologies are not standardized in implementation. Notwithstanding these inconsistencies amongst studies, certain pharmacotherapies have demonstrated beneficial effects on cognitive measures of individuals with MDD. A systematic review and meta-analysis that aimed to evaluate the overall effect of antidepressants on cognitive function in MDD revealed that SSRIs/SNRIs have more beneficial effects on memory domains compared to tricyclic antidepressants (TCAs), but exhibit equivalent effects on working memory compared to norepinephrine-dopamine reuptake inhibitors (NDRIs) [60]. Within the class of SSRIs, sertraline was found to have more beneficial effects on psychomotor speed compared to fluoxetine^60^. However, the results from this meta-analysis were limited by small sample sizes and large heterogeneity in cognitive testing which prevented pooling of effect sizes for a single domain. Improved standardization of cognitive assessment tools would be beneficial for future trials evaluating cognitive measures in MDD.

Duloxetine is an FDA-approved SNRI antidepressant medication. An imbalance or deficiency in serotonin and/or norepinephrine system function has been associated with cognitive deficits (68), providing the rationale for the use of SNRIs for the treatment of cognitive dysfunction in MDD. A study comparing cognitive function following either duloxetine or escitalopram treatment found that duloxetine resulted in greater improvements in declarative and working memory compared to escitalopram (69). Moreover, a double-blind, placebo-controlled trial involving elderly patients (ages 65 and older) with recurrent MDD found that 8-week treatment with duloxetine resulted in a significant improvement in composite measures of cognition compared to placebo. In this study, the composite cognitive score was mediated largely by improvement in verbal learning and memory (70). Duloxetine has also been assessed as a pro-cognitive antidepressant in young- to middle-aged subpopulations with MDD. For example, in a 12-week open-label trial, duloxetine was found to significantly improve cognitive function, particularly psychomotor speed (71).

Few studies have evaluated the pro-cognitive effects of other antidepressants for the treatment of cognitive dysfunction in MDD, with the exception of vortioxetine. Vortioxetine is an efficacious multimodal antidepressant that acts through a combination of serotonin reuptake inhibition and receptor activity. The efficacy of vortioxetine (10 or 20 mg/day) on cognition was evaluated in placebo-controlled study of adults between the ages of 18 and 65 with recurrent MDD and a current depressive episode (72). In this study, cognition was assessed using the DSST and RAVLT and significant improvements in objective measures of executive function, attention, and processing speed as well as learning and memory were described as a result of these assessments (72). The efficacy of vortioxetine in improving cognition in elderly patients (>65 years of age) with MDD has also been evaluated, and subsequently compared to the efficacy of duloxetine. Similar to the previous study, the RAVLT and DSST were used to evaluate cognitive performance. Significant improvements on the RAVLT were found in patients treated with duloxetine; however, significant improvements on both the RAVLT and DSST were found in patients treated with vortioxetine (73). The foregoing observation of differential effects on cognition could be mediated by the different mechanisms-of-action of the two drugs. Vortioxetine is a multimodal antidepressant hypothesized to exert its effects via its action on the serotonin reuptake transporter inhibition and cell surface serotonergiv receptors (e.g., 5HT3 and 5HT7). In comparison to duloxetine, the activity of vortioxetine as a 5-HT_1A_ receptor stimulator and 5-HT_3_ receptor antagonist may enhance cortical glutamatergic neuronal firing contributing to improved cognitive performance in individuals receiving vortioxetine treatment (73). A recent meta-analysis including nine placebo-controlled, randomized trials demonstrated that vortioxetine had the greatest effects on psychomotor speed, executive control, and cognitive control amongst all antidepressants evaluated for cognitive effects in placebo-controlled trials, whereas duloxetine had the greatest effect on delayed recall (74) Currently, vortioxetine is the only pharmacological agent that has been approved for the treatment of MDD through specific targeting of cognitive dysfunction (75).

As of May 2018, the U.S. FDA has announced that vortioxetine has demonstrated direct independent in clinically relevant improvements in cognitive dysfunction in adults 18–65 with MDD. The FDA recognizes significant improvement in cognitive function in vortioxetine-treated individuals as measured by the DSST when compared to placebo. Vortioxetine product insert update to include this information represents that first time any anti-depressant has an explicit mention regarding pro-cognition in MDD. The use of vortioxetine for the treatment of MDD has additionally been accepted by the European Medicines Agency.

Convergent evidence has implicated ketamine as a rapid-acting antidepressant in subpopulations with MDD that do not respond to conventional antidepressant therapies (76). Coincident with its use as an antidepressant in subanesthetic doses, it has been suggested that ketamine may also improve neurocognitive symptoms in TRD. Despite concerns regarding the effect of ketamine on cognition, replicated evidence in healthy controls has shown that ketamine does not impair recall for previously learned information(77, 78), and no impairments in executive function have been associated with ketamine treatment (79, 80). Additionally, in a subpopulation of TRD patients, ketamine was found to significantly reduce explicit suicidal ideation, as compared to the psychoactive placebo-control, midazolam (81). These findings suggest that ketamine treatment may exert beneficial effects on measures of executive functioning in patients with TRD. It is a testable hypothesis that the anti-suicide effects of ketamine are in part mediated by improvements in executive function (e.g., impulsivity) with ketamine treatment (82). However, the use of ketamine warrants further investigation for its application in the treatment of cognitive symptoms in patients with MDD. Early improvements in cognition have also been suggested to predict the efficacy of ketamine for TRD, implicating effects on cognition in the therapeutic mechanism of ketamine. In particular, lower levels of baseline neurocognitive performance in individuals with TRD were correlated with an increased antidepressant response to ketamine, as indicated by a ≥50% reduction in MADRS scores (83).

#### Additional Agents

Erythropoietin (EPO) is a glycoprotein secreted by the kidneys whose main function is to stimulate red blood cell production in the bone marrow (84). In addition to its hematopoietic role, EPO has been found to play a role in the central nervous system and is essential for neurodevelopment, adult neurogenesis and neuroprotection. Hippocampal EPO exerts neuroprotective and neurotrophic effects that have been demonstrated to enhance cognitive performance in various disease models. When systemically administered at therapeutic levels, EPO can cross the blood-brain barrier and enhance cognitive function in healthy animals (85). Due to its procognitive effects, EPO has been investigated as a treatment for the cognitive deficits observed in TRD. In double-blind placebo-controlled, parallel-group design study, subjects with clinically-defined unipolar TRD scoring ≥17 on the Hamilton Depression Rating Scale-17 (HAMD-17) were randomized to receive either EPO or saline infusions for 8 weeks. In this study, treatment with EPO significantly enhanced verbal recall and recognition compared to saline, which was maintained at follow-up at 14 weeks (86).

Aerobic/resistance exercise is also evidenced as a beneficial adjunctive therapeutic option for cognitive improvement in MDD (87). Replicated evidence implicates adjunctive exercise as an effective method for reducing depressive symptoms (88), with effects observed at levels commonly recommended for general public health (89). Studies have demonstrated that individuals with mild cognitive impairment who engage in regular exercise exhibit greater improvements in memory compared to those who do not engage in regular exercise (89). In addition, studies in healthy participants have demonstrated improvements in psychomotor speed, attention, visual memory, and spatial planning following engagement in exercise. One study found that 30-min exercise augmentation was associated with significant improvements in executive control processing (87). In a meta-analysis evaluating the effects of exercise on cognitive symptoms in MDD, the researchers found no significant procognitive effect of exercise (90); however, they did find that cognitive function was positively influenced by a combination of physical and cognitive activity as well as lower-intensity interventions with higher adherence rates (90). Of note, the findings of this meta-analysis may be limited by the quality of the data and methodological heterogeneity amongst studies. Continued research that stratifies participants by the type of exercise intervention and baseline characteristics (e.g., education level) is required to characterize the cognitive effects of exercise in depressed populations.

Intranasal insulin has also been investigated as a pro-cognitive agent in the treatment of mood and mental disorders. For example, cognitive performance in both Alzheimer's Disease (91) and Bipolar Disorder (92) has been shown to improve following treatment with intranasal insulin. Insulin availability and/or insulin receptor sensitivity has been implicated in MDD (93). Preclinical and clinical studies have demonstrated procognitive effects of intranasal insulin across multiple subdomains of cognition including learning and memory in both healthy and disease affected populations (91, 93). In a randomized, double-blind, placebo-controlled, crossover trial, although no significant improvements in neurocognitive function was observed with intranasal insulin treatment in individuals with MDD (94), the involvement of insulin receptors in cognitive and emotional processing warrants further investigation.

## Conclusion

Cognitive dysfunction is a core pathological feature of MDD that is often overlooked and under-evaluated in the diagnosis and treatment of the disorder. It serves as a principal mediator of psychosocial and functionality outcomes, with implications in workplace productivity and imminent return-to-work. Evaluation of both subjective and objective measures of cognition is imminent and relevant for improved functional outcomes in MDD.

Classical therapeutic approaches to MDD are insufficient, with poor existing response rates to first-, and even second-line antidepressant administration. While pharmacological treatment avenues have focused primarily on the recovery of mood symptoms, the evidence indicates that remitted patients continue to exhibit clinically-significant cognitive deficits that impact functional capacity. The inherent disconnect between mood remission and functional remission warrants the development of treatments that specifically target functionally-relevant domains (i.e., cognition). Current clinical paradigms have been insufficiently studied for their direct, independent, and clinically-significant effects on cognition. Vortioxetine is currently the only pharmacological agent approved for use in MDD that has been shown to exert direct and independent pro-cognitive effects. Disparate psychotherapeutic and adjunctive agents have been investigated from a precognitive perspective; however additional research is required to establish their independent efficacies. Based on the evidence, the development of therapeutic strategies that directly target cognitive symptoms, in addition to mood symptoms, in MDD is may be required for successful long-term remission and functional recovery in MDD.

## Author Contributions

HZ: substantial contributions to the conception and design of the manuscript. Drafting of the intellectual content of the work. ZP: substantial contributions to the conception of the manuscript. CP: drafting of the intellectual content of the work. EB: drafting of the intellectual content of the work. NM: critical revision of the work for content. AS: critical revision of the work for content. MI: critical revision of the work, and assistance in the design of figures. SY: critical revision of the work. LL: critical revision of the work. CR: critical revision of the work. RM: substantial contributions to the conception and design of the manuscript, critical revision of the work.

### Conflict of Interest Statement

EB has received honoraria as speaker and advisory board member of Daiichy-Sankyo. RM is a consultant to speak on behalf for, and/or has received research support from Allergan, AstraZeneca, Bayer, Bristol-Myers, Squibb, Janssen-Ortho, Eli Lilly, Lundbeck, Merck, Otsuka, Pfizer, Sunovion, Neurocrine, and Takeda. The remaining authors declare that the research was conducted in the absence of any commercial or financial relationships that could be construed as a potential conflict of interest.

## References

[1] World Health Organization Depression and Other Common Mental Disorders Global Health Estimates. Geneva: World Health Organization (2017).

[2] Carvalho AF Miskowiak KK Hyphantis TN Kohler CA Alves GS Bortolato B.. Cognitive dysfunction in depression - pathophysiology and novel targets. CNS Neurol Disord. Drug Targets (2014) 13:1819–35. 10.2174/1871527313666141130203627 25470397

[3] Trivedi MH Rush AJ Wisniewski SR Nierenberg AA Warden D Ritz L. Evaluation of outcomes with citalopram for depression using measurement-based care in STAR^*^D: Implications for Clinical Practice. Am J Psychiatry (2006) 163:28–40. 10.1176/appi.ajp.163.1.28 16390886

[4] Stotland NL. Recovery from depression. Psychiatric Clin North Am. (2012) 35:37–49. 10.1016/j.psc.2011.11.007 22370489

[5] Jacobs P Ohinmaa A Escober-Doran C Patterson S Slomp M PMH31 measuring the economic burden of depression using patient records. Value Health (2009) 12:A356 10.1016/S1098-3015(10)74750-1

[6] Lépine J-P Briley M. The increasing burden of depression. Neuropsychiatric Dis Treatmen (2011) 7:3–7. 10.2147/NDT.S19617 21750622 PMC3131101

[7] Gorwood P Corruble E Falissard B Goodwin GM. Toxic effects of depression on brain function: Impairment of delayed recall and the cumulative length of depressive disorder in a large sample of depressed outpatients. Am J Psychiatry (2008) 165:731–739. 10.1176/appi.ajp.2008.07040574 18381906

[8] Zimmerman M McGlinchey JB Posternak MA Friedman M Boerescu D Attiullah N (2006). Discordance between self-reported symptom severity and psychosocial functioning ratings in depressed outpatients: implications for how remission from depression should be defined. Psychiatry Res. (2006) 141:185–91. 10.1016/j.psychres.2005.05.016 16499976

[9] Buist-Bouwman MA Ormel J de Graaf R de Jonge P van Sonderen E Alonso J and ESEMeD/MHEDEA 2000 investigators the Esem. 2000. Mediators of the association between depression and role functioning. Acta Psychiatrica Scand. (2008) 118:451–8. 10.1111/j.1600-0447.2008.01285.x 18853945 PMC3659780

[10] Gonda X Pompili M Serafini G Carvalho AF Rihmer Z Dome P. The role of cognitive dysfunction in the symptoms and remission from depression. Anna Gener Psychiatry (2015) 14:27. 10.1186/s12991-015-0068-9 26396586 PMC4578787

[11] Jaeger J Berns S Uzelac S Davis-Conway S. Neurocognitive deficits and disability in major depressive disorder. Psychiatry Res. (2006) 145:39–48. 10.1016/j.psychres.2005.11.011 17045658

[12] McIntyre RS Cha DS Soczynska JK Woldeyohannes HO Gallaugher LA Kudlow P. Cognitive deficits and functional outcomes in major depressive disorder: determinants, substrates, and treatment interventions. Depress Anxiety (2013) 30:515–27. 10.1002/da.22063 23468126

[13] Stedman TL Stedman's Medical Dictionary for the Health Professions and Nursing. Holland: Wolters Kluwer Health/Lippincott Williams & Wilkins (2012).

[14] McIntyre RS Xiao HX Syeda K Vinberg M Carvalho AF Mansur RB. The Prevalence, measurement, and treatment of the cognitive dimension/domain in major depressive disorder. CNS Drugs (2015) 29:577–89. 10.1007/s40263-015-0263-x 26290264

[15] Depressive Disorders In Diagnostic and Statistical Manual of Mental Disorders. Washington, DC: American Psychiatric Association (2013). 10.1176/appi.books.9780890425596.dsm04

[16] Bora E Harrison BJ Yücel M Pantelis C. Cognitive impairment in euthymic major depressive disorder: a meta-analysis. Psychol Med. (2013) 43:2017–26. 10.1017/S0033291712002085 23098294

[17] Harrison JE Lam RW Baune BT McIntyre RS. Selection of cognitive tests for trials of therapeutic agents. Lancet Psychiatry (2016) 3:499. 10.1016/S2215-0366(16)30067-0 27262042

[18] Niciu MJ Mathews DC Nugent AC Ionescu DF Furey ML Richards EM. Developing biomarkers in mood disorders research through the use of rapid-acting Antidepressants. Depress Anxiety (2014) 31:297–307. 10.1002/da.22224 24353110 PMC3984598

[19] Insel TR. The NIMH research domain criteria (RDoC) project: precision medicine for psychiatry. Am J Psychiatry (2014) 171:395–7. 10.1176/appi.ajp.2014.14020138 24687194

[20] Roiser JP Sahakian BJ. Hot and cold cognition in depression. CNS Spectrums (2013) 18:139–49. 10.1017/S1092852913000072 23481353

[21] Fossati P Hevenor Simon J Graham SJ Grady C Keightley ML Fergus Craik M Mayberg H. In search of the emotional Self: an fMRI study using positive and negative emotional words. Article Am J Psychiatry (1938) 160:1938–45 14594739 10.1176/appi.ajp.160.11.1938

[22] Clark L Chamberlain SR Sahakian BJ. Neurocognitive Mechanisms in Depression: Implications for Treatment. Annu Rev Neurosci. (2009) 32:57–74. 10.1146/annurev.neuro.31.060407.125618 19400725

[23] Kessing LV Andersen PK. Evidence for clinical progression of unipolar and bipolar disorders. Acta Psychiatrica Scand. (2017) 135:51–64. 10.1111/acps.12667 27858964

[24] Cserjési R Luminet O Poncelet A.-S Lénárd L. Altered executive function in obesity. exploration of the role of affective states on cognitive abilities. Appetite. (2009) 52:535–9. 10.1016/j.appet.2009.01.003 19260167

[25] Larochette A-C Harrison AG Rosenblum Y Bowie CR. Additive Neurocognitive Deficits in Adults with attention-deficit/hyperactivity disorder and depressive symptoms. Arch Clin Neuropsychol. (2011) 26:385–95. 10.1093/arclin/acr033 21586538

[26] Mansur RB Cha DS Woldeyohannes HO Soczynska JK Zugman A Brietzke E. Diabetes mellitus and disturbances in brain connectivity: abidirectional relationship? Neuromol Med. (2014) 16:658–68. 10.1007/s12017-014-8316-8 24974228

[27] Thomas AJ Gallagher P Robinson LJ Porter RJ Young AH Ferrier IN. A comparison of neurocognitive impairment in younger and older adults with major depression. Psychol Med. (2009) 39:725. 10.1017/S0033291708004042 18667097

[28] Herrmann LL Le Masurier M Ebmeier KP. White matter hyperintensities in late life depression: a systematic review. J Neurol Neurosurg Psychiatry (2007) 79:619–24. 10.1136/jnnp.2007.124651 17717021

[29] Gildengers AG Butters MA Chisholm D Anderson SJ Begley A Holm M. Cognition in older adults with bipolar disorder versus major depressive disorder. Bipolar Disord. (2012) 14:198–205. 10.1111/j.1399-5618.2012.00995.x 22420595 PMC3379872

[30] Withall A Harris LM Cumming SR. A longitudinal study of cognitive function in melancholic and non-melancholic subtypes of major depressive disorder. J Affect Disord. (2010) 123:150–7. 10.1016/j.jad.2009.07.012 19698995

[31] Pan Z Grovu RC Cha DS Carmona NE Subramaniapillai M Shekotikhina M. Pharmacological treatment of cognitive symptoms in major depressive disorder. CNS Neurol Disord. - Drug Targets (2017) 16:1–9. 10.2174/1871527316666170919115100 28933261

[32] Herrera-Guzmán I Gudayol-Ferré E Herrera-Guzmán D Guàrdia-Olmos J Hinojosa-Calvo E Herrera-Abarca JE. Effects of selective serotonin reuptake and dual serotonergic–noradrenergic reuptake treatments on memory and mental processing speed in patients with major depressive disorder. J Psychiatric Res. (2009) 43:855–63. 10.1016/j.jpsychires.2008.10.015 19128810

[33] Lemos-Miller A Kearney CA. Depression and ethnicity as intermediary variables among dissociation, trauma-related cognitions, and PTSD Symptomatology in Youths. J Nervous Mental Dis. (2006) 194:584–90. 10.1097/01.nmd.0000230407.12687.ba 16909066

[34] Airaksinen E Wahlin Å Forsell Y Larsson M. Low episodic memory performance as a premorbid marker of depression: evidence from a 3-year follow-up. Acta Psychiatrica Scand. (2007) 115:458–65. 10.1111/j.1600-0447.2006.00932.x 17498157

[35] Simons CJP Jacobs N Derom C Thiery E Jolles J Van Os J. Cognition as predictor of current and follow-up depressive symptoms in the general population. (2009) 120:45–52. 10.1111/j.1600-0447.2008.01339.x 19133876

[36] Park C Pan Z Brietzke E Subramaniapillai M Rosenblat JD Zuckerman H. Predicting antidepressant response using early changes in cognition: a systematic review. Behav Brain Res. (2018) 353:154–60. 10.1016/j.bbr.2018.07.011 30031025

[37] Gualtieri CT Morgan DW. The frequency of cognitive impairment in patients with anxiety, depression, and bipolar disorder: an unaccounted source of variance in clinical trials. J Clin Psychiatry (2008) 69:1122–30. 18572982 10.4088/jcp.v69n0712

[38] Papakostas GI. Cognitive symptoms in patients with major depressive disorder and their implications for clinical practice. J Clin Psychiatry (2014) 75:8–14. 10.4088/JCP.13r08710 24345473

[39] McIntyre RS Lee Y Cognition in major depressive disorder: a “systemically important functional index” (SIFI). Curr Opin Psychiatry (2016) 29:48–55. 10.1097/YCO.0000000000000221 26575300

[40] Birnbaum HG Kessler RC Kelley D Ben-Hamadi R Joish VN Greenberg PE. Employer burden of mild, moderate, and severe major depressive disorder: mental health services utilization and costs, and work performance. Depress Anxiety (2010) 27:78–89. 10.1002/da.20580 19569060

[41] McIntyre RS Soczynska JZ Woldeyohannes HO Alsuwaidan MT Cha DS Carvalho AF. The impact of cognitive impairment on perceived workforce performance: results from the international mood disorders collaborative project. Comprehens Psychiatry (2015) 56:279–82. 10.1016/j.comppsych.2014.08.051 25439523

[42] Preiss M Kucerova H Lukavsky J Stepankova H Sos P Kawaciukova R. Cognitive deficits in the euthymic phase of unipolar depression. Psychiatry Res. (2009) 169:235–9. 10.1016/j.psychres.2008.06.042 19765829

[43] Hasselbalch BJ Knorr U Kessing LV. Cognitive impairment in the remitted state of unipolar depressive disorder: a systematic review. J Affect Disord. (2011) 134:20–31. 10.1016/J.JAD.2010.11.011 21163534

[44] Paelecke-Habermann Y Pohl J Leplow B. Attention and executive functions in remitted major depression patients. J Affect Disord. (2005) 89:125–35. 10.1016/j.jad.2005.09.006 16324752

[45] McIntyre RS Best M Bowie C Carmona N Cha D Lee Y. The THINC-integrated tool (THINC-it) screening assessment for cognitive dysfunction: validation in patients with major depressive disorder. J Clin Psychiatry (2017) 78:873–81. 10.4088/JCP.14m09658 28858441

[46] Ott CV Bjertrup AJ Jensen JH Ullum H Sjælland R Purdon SE. Screening for cognitive dysfunction in unipolar depression: validation and evaluation of objective and subjective tools. J Affect Disord. (2016) 190:607–15. 10.1016/j.jad.2015.10.059 26583350

[47] Mackin RS Insel P Tosun D Mueller SG Schuff N Truran-Sacrey D. The effect of subsyndromal symptoms of depression and white matter lesions on disability for individuals with mild cognitive impairment. Am J Geriat Psychiatry (2013) 21:906–14. 10.1016/j.jagp.2013.01.021 23567388 PMC5548455

[48] Bowie CR Gupta M Holshausen K Jokic R Best M Milev R. Cognitive remediation for treatment-resistant depression. J Nervous Mental Dis. (2013) 201:680–5. 10.1097/NMD.0b013e31829c5030 23896849

[49] Drevets WC Price JL Furey ML. Brain structural and functional abnormalities in mood disorders: implications for neurocircuitry models of depression. Brain Struct Funct. (2008) 213:93–118. 10.1007/s00429-008-0189-x 18704495 PMC2522333

[50] Porter RJ Bowie CR Jordan J Malhi GS. Cognitive remediation as a treatment for major depression: a rationale, review of evidence and recommendations for future research. Aust N Zeal J Psychiatry (2013) 47:1165–75. 10.1177/0004867413502090 23956342

[51] Naismith SL Redoblado-Hodge MA Lewis SJG Scott EM Hickie IB. Cognitive training in affective disorders improves memory: a preliminary study using the NEAR approach. J Affect Disord. (2010) 121:258–62. 10.1016/J.JAD.2009.06.028 19616856

[52] Lee RSC Redoblado-Hodge MA Naismith SL Hermens DF Porter MA Hickie IB. Cognitive remediation improves memory and psychosocial functioning in first-episode psychiatric out-patients. Psychol Med. (2013) 43:1161–73. 10.1017/S0033291712002127 23237010 PMC3642720

[53] Naismith SL Diamond K Carter PE Norrie LM Redoblado-Hodge MA Lewis SJG. Enhancing memory in late-life depression: the effects of a combined psychoeducation and cognitive training program. Am J Geriatr Psychiatry (2011) 19:240–8. 10.1097/JGP.0b013e3181dba587 20808114

[54] Cuijpers P Berking M Andersson G Quigley L Kleiboer A Dobson KS A meta-analysis of cognitive-behavioural therapy for adult depression, alone and in comparison with other treatments. Can J Psychiatry (2013) 5858:376–85. 10.1177/070674371305800702 23870719

[55] DeRubeis RJ Hollon SD Amsterdam JD Shelton RC Young PR Salomon RM. Cognitive therapy vs medications in the treatment of moderate to severe depression. Arch Gener Psychiatry (2005) 62:409. 10.1001/archpsyc.62.4.409 15809408

[56] Lemogne C Delaveau P Freton M Guionnet S Fossati P. Medial prefrontal cortex and the self in major depression. J Affect Disord. (2012) 136:e1–1. 10.1016/j.jad.2010.11.034 21185083

[57] Yoshimura S Okamoto Y Onoda K Matsunaga M Ueda K Suzuki S Yamawaki S. Rostral anterior cingulate cortex activity mediates the relationship between the depressive symptoms and the medial prefrontal cortex activity. J Affect Disord. (2010) 122:76–85. 10.1016/j.jad.2009.06.017 19589603

[58] Yoshimura S Okamoto Y Matsunaga M Onoda K Okada G Kunisato Y. Cognitive behavioral therapy changes functional connectivity between medial prefrontal and anterior cingulate cortices. J Affect Disord. (2017) 208:610–4. 10.1016/J.JAD.2016.10.017 27810274

[59] Semkovska M Mcloughlin DM. Objective cognitive performance associated with electroconvulsive therapy for depression: a systematic review and meta-analysis. (2010) 68:568–77. 10.1016/j.biopsych.2010.06.009 20673880

[60] Mathews A MacLeod C. Cognitive vulnerability to emotional disorders. Ann Rev Clin Psychol. (2005) 1:167–95. 10.1146/annurev.clinpsy.1.102803.143916 17716086

[61] Miskowiak KW Carvalho AF. “Hot” cognition in major depressive disorder: a systematic review. CNS Neurol Disord Drug Targets (2014) 13:1787–803. 10.2174/1871527313666141130205713 25470389

[62] Miskowiak K Papadatou-Pastou M Cowen PJ Goodwin GM Norbury R Harmer CJ. Single dose antidepressant administration modulates the neural processing of self-referent personality trait words. NeuroImage (2007) 37:904–11. 10.1016/j.neuroimage.2007.05.036 17625917

[63] Nathan PJ Phan KL Harmer CJ Mehta MA Bullmore ET. Increasing pharmacological knowledge about human neurological and psychiatric disorders through functional neuroimaging and its application in drug discovery. Curr Opin Pharmacol. (2014) 14:54–61. 10.1016/j.coph.2013.11.009 24565013

[64] Miskowiak KW Kessing LV Ott CV Macoveanu J Harmer CJ Jørgensen A. Does a single session of electroconvulsive therapy alter the neural response to emotional faces in depression? A randomised sham-controlled functional magnetic resonance imaging study. J Psychopharmacol. (2017) 31:1215–24. 10.1177/0269881117699615 28351201

[65] Rasmussen KG. Some considerations in choosing electroconvulsive therapy versus transcranial magnetic stimulation for depression. J ECT (2011) 27:51–4. 10.1097/YCT.0b013e3181da84c6 21343711

[66] Serafini G Pompili M Murri MB Respino M Ghio L Girardi P. The effects of repetitive transcranial magnetic stimulation on cognitive performance in treatment-resistant depression. a systematic review. Neuropsychobiology (2015) 71:125–39. 10.1159/000381351 25925699

[67] Pascual-Leone A Rubio B Pallard ó F Catalá MD. Rapid-rate transcranial magnetic stimulation of left dorsolateral prefrontal cortex in drug-resistant depression. Lancet (1996) 348:233–237. 8684201 10.1016/s0140-6736(96)01219-6

[68] Ressler KJ Nemeroff CB. Role of norepinephrine in the pathophysiology of neuropsychiatric disorders. CNS Spectrums. (2001) 6:663–70. 10.1017/S1092852900001358 15520614

[69] Herrera-Guzmán I Herrera-Abarca JE Gudayol-Ferré E Herrera-Guzmán D Gómez-Carbajal L Peña-Olvira M. Effects of selective serotonin reuptake and dual serotonergic–noradrenergic reuptake treatments on attention and executive functions in patients with major depressive disorder. Psychiatry Res. (2010) 177:323–9. 10.1016/j.psychres.2010.03.006 20385412

[70] Raskin J Wiltse CG Siegal A Sheikh J Xu J Dinkel JJ. Efficacy of duloxetine on cognition, depression, and pain in elderly patients with major depressive disorder: an 8-week, double-blind, placebo-controlled trial. Am J Psychiatry (2007) 164:900–9. 10.1176/ajp.2007.164.6.900 17541049

[71] Greer TL Sunderajan P Grannemann BD Kurian BT Trivedi MH. Does duloxetine improve cognitive function independently of its antidepressant effect in patients with major depressive disorder and subjective reports of cognitive dysfunction? Depres Res Treatment (2014) 2014:627863. 10.1155/2014/627863 24563781 PMC3915915

[72] McIntyre RS Lophaven S Olsen CK. A randomized, double-blind, placebo-controlled study of vortioxetine on cognitive function in depressed adults. Int J Neuropsychopharmacol. (2014) 17:1557–67. 10.1017/S1461145714000546 24787143 PMC4162519

[73] Katona C Hansen T Kurre Olsen C Cornelius Katona C. A randomized, double-blind, placebo-controlled, duloxetine-referenced, fixed-dose study comparing the efficacy and safety of Lu AA21004 in elderly patients with major depressive disorder. Int Clin Psychopharmacol. (2012) 27:15–23. 10.1097/YIC.0b013e3283542457 22572889

[74] Rosenblat JD Kakar R McIntyre RS. The cognitive effects of antidepressants in major depressive disorder: a systematic review and meta-analysis of randomized clinical trials. Int J Neuropsychopharmacol. (2015) 19:1–13. 10.1093/ijnp/pyv082 26209859 PMC4772818

[75] Lundbeck FDA Updates Trintellix® (vortioxetine) Label to Include Data Showing Improvement in Processing Speed, an Important Aspect of Cognitive Function in acute Major Depressive Disorder (MDD). (2018). Available online at: https://globenewswire.com/news-release/2018/05/02/1495434/0/en/FDA-updates-Trintellix-vortioxetine-label-to-include-data-showing-improvement-in-processing-speed-an-important-aspect-of-cognitive-function-in-acute-Major-Depressive-Disorder-MDD.html (Accessed May 17, 2018)

[76] Coyle CM Laws KR. The use of ketamine as an antidepressant: a systematic review and meta-analysis. Human Psychopharmacol. (2015) 30:152–63. 10.1002/hup.2475 25847818

[77] Perry EB Cramer JA Cho H-S Petrakis IL Karper LP Genovese A. Psychiatric safety of ketamine in psychopharmacology research. Psychopharmacology. (2007) 192:253–60. 10.1007/s00213-007-0706-2 17458544

[78] Rowland LM Astur RS Jung RE Bustillo JR Lauriello J Yeo RA. Selective cognitive impairments associated with NMDA receptor blockade in humans. Neuropsychopharmacology (2005) 30:633–9. 10.1038/sj.npp.1300642 15647751

[79] Krystal JH D'Souza DC Karper LP Bennett A Abi-Dargham A Abi-Saab D. Interactive effects of subanesthetic ketamine and haloperidol in healthy humans. Psychopharmacology (1999) 145:193–204. 10463321 10.1007/s002130051049

[80] Morgan CJA Mofeez A Brandner B Bromley L Curran HV. Acute effects of ketamine on memory systems and psychotic symptoms in healthy volunteers. Neuropsychopharmacology. (2004) 29:208–18. 10.1038/sj.npp.1300342 14603267

[81] Price RB Iosifescu DV Murrough JW Chang LC Al Jurdi RK Iqbal SZ. Effects of ketamine on explicit and implicit suicidal cognition: a randomized controlled trial in treatment-resistant depression. Depress Anxiety (2014) 31:335–43. 10.1002/da.22253 24668760 PMC4112410

[82] Lee Y Syeda K Maruschak NA Cha DS Mansur RB Wium-Andersen IK. A new perspective on the anti-suicide effects with ketamine treatment: a procognitive effect. J Clin Psychopharmacol. (2016) 36:50–6. 10.1097/JCP.0000000000000441 26658082

[83] Rong C Park C Rosenblat JD Subramaniapillai M Zuckerman H Fus D. Predictors of response to ketamine in treatment resistant major depressive disorder and bipolar disorder. Int J Environ Res Public Health (2018) 15:771. 10.3390/ijerph15040771 29673146 PMC5923813

[84] Bunn HF. Erythropoietin. Cold Spring Harbor Perspect Med. (2013) 3:a011619. 10.1101/cshperspect.a011619 23457296 PMC3579209

[85] Miskowiak KW Vinberg M Harmer CJ Ehrenreich H Kessing LV. Erythropoietin: a candidate treatment for mood symptoms and memory dysfunction in depression. Psychopharmacology (2012) 219:687–98. 10.1007/s00213-011-2511-1 21947319

[86] Miskowiak KW Vinberg M Christensen EM Bukh JD Harmer CJ Ehrenreich H. Recombinant human erythropoietin for treating treatment-resistant depression: a double-blind, randomized, placebo-controlled phase 2 trial. Neuropsychopharmacology (2014) 39:1399–408. 10.1038/npp.2013.335 24322509 PMC3988543

[87] Kubesch S Bretschneider V Freudenmann R Weidenhammer N Lehmann M Spitzer M. Aerobic endurance exercise improves executive functions in depressed patients. J Clin Psychiatry (2003) 64:1005–12. 10.4088/JCP.v64n0905 14628975

[88] Callaghan P Khalil E Morres I Carter T. Pragmatic randomised controlled trial of preferred intensity exercise in women living with depression. BMC Public Health (2011) 11:465. 10.1186/1471-2458-11-465 21663696 PMC3128029

[89] Stanton R Reaburn P. Exercise and the treatment of depression: A review of the exercise program variables. J Sci Med Sport (2014) 17:177–82. 10.1016/j.jsams.2013.03.010 23602562

[90] Sun M Lanctot K Herrmann N Gallagher D. Exercise for cognitive symptoms in depression: a systematic review of interventional studies. Can J Psychiatry (2018) 63:115–28. 10.1177/0706743717738493 29186973 PMC5788135

[91] Reger MA Watson GS Green PS Wilkinson CW Baker LD Cholerton B. Intranasal insulin improves cognition and modulates -amyloid in early AD. Neurology. (2008) 70:440–8. 10.1212/01.WNL.0000265401.62434.36 17942819

[92] McIntyre RS Soczynska JK Woldeyohannes HO Miranda A Vaccarino A MacQueen G. A randomized, double-blind, controlled trial evaluating the effect of intranasal insulin on neurocognitive function in euthymic patients with bipolar disorder. Bipolar Disord. (2012) 14:697–706. 10.1111/bdi.12006 23107220

[93] Craft S Baker LD Montine TJ Minoshima S Watson GS Claxton A. Intranasal insulin therapy for Alzheimer disease and amnestic mild cognitive impairment: a pilot clinical trial. Archi Neurol. (2012) 69:29–38. 10.1001/archneurol.2011.233 21911655 PMC3260944

[94] Cha DS Best MW Bowie CR Gallaugher LA Woldeyohannes HO Soczynska JK. A randomized, double-blind, placebo-controlled, crossover trial evaluating the effect of intranasal insulin on cognition and mood in individuals with treatment-resistant major depressive disorder. J Affect Disord. (2017) 210:57–65. 10.1016/j.jad.2016.12.006 28013123

